# Site-Specific Labeling of Neurotrophins and Their Receptors via Short and Versatile Peptide Tags

**DOI:** 10.1371/journal.pone.0113708

**Published:** 2014-11-26

**Authors:** Laura Marchetti, Teresa De Nadai, Fulvio Bonsignore, Mariantonietta Calvello, Giovanni Signore, Alessandro Viegi, Fabio Beltram, Stefano Luin, Antonino Cattaneo

**Affiliations:** 1 NEST, Scuola Normale Superiore and Istituto Nanoscienze – CNR, Pisa, Italy; 2 BioSNS Laboratory, Scuola Normale Superiore and Istituto di Neuroscienze - CNR, Pisa, Italy; 3 IIT@NEST, Center for Nanotechnology Innovation, Pisa, Italy; University of Naples Federico II, Italy

## Abstract

We present a toolbox for the study of molecular interactions occurring between NGF and its receptors. By means of a suitable insertional mutagenesis method we show the insertion of an 8 amino acid tag (A4) into the sequence of NGF and of 12 amino acid tags (A1 and S6) into the sequence of TrkA and P75NTR NGF-receptors. These tags are shortened versions of the acyl and peptidyl carrier proteins; they are here covalently conjugated to the biotin-substituted arm of a coenzyme A (coA) substrate by phosphopantetheinyl transferase enzymes (PPTases). We demonstrate site-specific biotinylation of the purified recombinant tagged neurotrophin, in both the immature proNGF and mature NGF forms. The resulting tagged NGF is fully functional: it can signal and promote PC12 cells differentiation similarly to recombinant wild-type NGF. Furthermore, we show that the insertion of A1 and S6 tags into human TrkA and P75NTR sequences leads to the site-specific biotinylation of these receptors at the cell surface of living cells. Crucially, the two tags are labeled selectively by two different PPTases: this is exploited to reach orthogonal fluorolabeling of the two receptors co-expressed at low density in living cells. We describe the protocols to obtain the enzymatic, site-specific biotinylation of neurotrophins and their receptors as an alternative to their chemical, nonspecific biotinylation. The present strategy has three main advantages: i) it yields precise control of stoichiometry and site of biotin conjugation; ii) the tags used can be functionalized with virtually any small probe that can be carried by coA substrates, besides (and in addition to) biotin; iii) above all it makes possible to image and track interacting molecules at the single-molecule level in living systems.

## Introduction

Neurotrophic factors, whose prototype member is nerve growth factor (NGF) [1], are a family of secreted proteins that crucially regulate neuronal development, survival and plasticity both in the central and in the peripheral nervous system. Their biological activity stems largely from the binding of two membrane receptor types: the tropomyosin receptor kinase (Trk) family and the p75 neurotrophin (P75NTR) coreceptor [2]. While most of the signaling cascades activated by NGF binding to TrkA and P75NTR receptors were identified, the impact on receptor dynamics caused by TrkA and P75NTR engagement by NGF and the regulation of their cellular traffic are far from being understood. In this context, techniques that make it possible to investigate the TrkA-NGF-P75NTR dynamic interplay in a physiological context (e.g. the intact plasma membrane and endosomes in living neuronal cells) can be decisive to unveil the molecular mechanisms governing their functional interactions. To date these issues were poorly explored largely owing to the lack of suitable experimental tools. Indeed these studies require to selectively label neurotrophins and their receptors in order to simultaneously detect them in living cells as three independent signals. Ideally, labeling strategies should have the following properties: i) if labeling relies on the use of tagged constructs of the proteins of interest, tags should be as small as possible, in order to minimally interfere with protein functionality and with the formation of molecular complexes; ii) a 1∶1 stoichiometry between the labeled protein and the probe should be obtained; iii) they should be versatile, in order to yield molecular species that can be derivatized with different probes, depending on the experimental needs (*e.g.* biotin, fluorophores, gold or magnetic nanoparticles); iv) they should allow the simultaneous differential labeling of at least two molecules that are supposed to form a complex (i.e. neurotrophin and one of its receptors, or two neurotrophin receptors).

We previously demonstrated that the insertion of the acyl carrier protein (ACP) tag [3] at the extracellular domain of TrkA makes it possible to specifically label the receptor at the cell surface when the construct is transfected in living cells [4], [5]. The ACP tag belongs to a family of protein and peptide tags, which can be covalently conjugated to virtually any small-probe substituted phosphopantetheinyl (PP) arm of Coenzyme A (CoA) substrate by post-translational modification enzymes named PP transferases (PPTases) [6]. The ACP tag was shown not to interfere with TrkA receptor function [4]. When coupled to various fluorescent probes, this tool made it possible to monitor in living cells single TrkA movements and changes of oligomerization state upon binding of different biologically-relevant ligands including NGF and proNGF [5].

Here we demonstrate the insertion of an 8-amino-acids tag into the sequence of NGF and of 12-amino-acids tags into the sequence of the two NGF receptors, TrkA and P75NTR. These tags derive from *in vitro* evolution studies [7], [8] committed to the shortening of the ACP and peptidyl carrier protein (PCP) [9] tags. The tags were inserted by an insertional mutagenesis method, based on a modification of the standard site-directed mutagenesis protocol, that allows their insertion in the protein of interest with no need for any additional linker sequence and thus with minimal interference with protein activity. Here we conjugate biotin to the tags by using PPTases in the presence of CoA-biotin substrates. We demonstrate that upon insertion of these tags, a site-specific biotinylation is achieved for the purified recombinant neurotrophin (both immature proNGF and mature NGF forms) and for TrkA and P75NTR receptors expressed in the membrane of living cells. Moreover, the properties of orthogonal labeling displayed by the 12-amino-acid A1 and S6 tags [7] are exploited to simultaneously label single TrkA and P75NTR receptors with two spectrally-distinct fluorophores, even when the two receptors are expressed in the same cell. We show that the insertion of such tags in the chosen sites has no measurable impact in NGF functionality or in the correct translocation of TrkA and P75NTR receptors at the cell membrane. Application of these three nanoprobes to the investigation of trafficking and interactions of NGF and its receptors in living cells will be discussed.

## Materials and Methods

### Plasmids preparation

Human TrkA and P75NTR cDNAs cloned in frame to EGFP in pReceiver-M03 (OmicsLink, ImaGenes Berlin) and human proNGF cDNA cloned in pET11 vector [10] were used as templates. The cDNA coding sequences of A1 (GDSLDMLEWSLM) and S6 (GDSLSWLLRLLN) tags were inserted into TrkA and P75NTR downstream the N-terminal signal peptide of localization at the cell membrane. The cDNA coding sequence of A4 (DSLDMLEW) was inserted at the C-terminus of proNGF. We used the QuickChange XL Site-Directed Mutagenesis kit (Agilent Technologies) with modifications adapted from what previously reported in [11]. Briefly, we followed the scheme of a standard site-directed mutagenesis, except for two main modifications: i) the use of a two-step PCR program (Table S2) in which during the first step amplifications with forward and reverse primers were kept separate; ii) the splitting of the amino acids to be introduced in two sequential PCR reactions. All experimental details of this protocol are provided in Text S1. In order to remove the EGFP sequence from the resulting A1/S6-TrkA and A1/S6-P75NTR constructs, their full-length cDNA were further PCR amplified using FW and RV primers bearing MluI restriction sites; the amplification products were subcloned into pCR2.1TA cloning vector (Invitrogen), before final insertion into the MluI sites of the TMPrtTA “all-in-one” inducible lentiviral vector [12]. The ACP-TrkA construct used as a control was described in [4].

### proNGF and NGF expression and purification

Tagged proNGF–A4 was expressed in *E. coli* and purified according to the protocols published for the purification of recombinant human proNGF [13], [14]. Briefly, ion exchange FPLC chromatography was used for the purification of proNGF after its extraction from inclusion bodies and subsequent pulsed refolding. NGF-A4 was further obtained from controlled proteolytic cleavage of proNGF-A4 by trypsin (typically, 1 µg enzyme was incubated with 250 µg proneurotrophin for up to 15 h at 4°C to achieve exhaustive protein digestion). NGF-A4 was purified from the digestion reaction by ion exchange FPLC chromatography. We calculated the production yields of the purified proteins as the quantity of obtained protein (in mg) per liter of bacterial culture volume (Fig. S1). We found the yield of proNGF-A4 production to be ∼30% of that typically obtained for wt proNGF. Mature NGF-A4 production yield was found to be ∼15% of that obtained for wt NGF. All steps above described were checked by SDS-PAGE and Coomassie staining.

### Cell culture

PC12 (ATCC, CRL-1721) cells were maintained at 37°C, 5% CO_2_ in RPMI1640 medium supplemented with 10% horse serum, 5% fetal bovine serum and 1% penicillin/streptomycin (Gibco). PC12 differentiation was induced by treatment with ∼50 ng/ml wt NGF (Alomone Labs), recombinant NGF-A4 or biotinylated NGF-A4 (NGF-A4b); cells were observed after five days at a Leica DM6000 microscope capable of transmission DIC imaging. Morphometric analysis of PC12 differentiation was performed on imaged cells, by measuring two parameters (Fig. S2): the percentage (%) of PC12 differentiation (*Diff*), as obtained by counting the number of cells displaying at least one neurite with a length equal to, or greater than, the diameter of the cell body and expressing it as a percentage of the total number of cells in a field [15]; the average number of neurites per differentiated cell (av. neurites/cell) in a field. Statistical analysis was carried out using the one-way ANOVA test and Bonferroni's comparison of means, with P<0.05 considered as significant. SH-SY5Y cells (ECACC, 94030304) were cultured at 37°C, 5% CO_2_ in DMEM-F12 medium supplemented with 10% fetal bovine serum and 1% penicillin/streptomycin (Gibco). SH-SY5Y transfection was performed with Lipofectamine 2000 (Invitrogen) according to the manufacturers' instructions.

### Synthesis of CoA-biotin conjugate

All reactions were carried out under nitrogen. Solvents (Romil) were of ultra-pure, anhydrous grade, and were used without further purifications. Reagents (Aldrich) were used as received. PBS was freshly degassed by sonication under vacuum. Chromatographic analyses were performed using a Phenomenex Fusion 150×4.6 mm column on a Dionex Ultimate 3000 HPLC equipped with PDA detector and fraction collector and interfaced with an ABSciex API 3200 Q-TRAP mass spectrometer. HPLC solvents: Ammonium formate 5 mM (eluent A)/acetonitrile (eluent B). CoA-biotin conjugate was prepared with a two-step approach:

1. *Conjugation of aminoethyl maleimide with biotin:* Biotin (10 mg) was dissolved in DMF (250 µL). NHS (1.1 eq) and 2-aminoethylmaleimide (1.1 eq) dissolved in DMF (100 µL) were added to the solution. EDC (1.2 eq) dissolved in PBS (100 µL) was added and the solution stirred 4 h at 25°C. When complete conversion of the reagent was achieved (HPLC) the product was purified by semipreparative RP-HPLC (Column: Phenomenex Proteo 250×9.6 mm) and freeze-dried. MS (ESI): Predicted (m/z): [M+H]^+^: 367.14. Found: 367.3. MS parameters: Curtain gas 10 L/min; Ion spray voltage: 5500 V; Temperature: not used; Declustering potential: 15 V; Entrance potential: 10 V; Collision energy: 35 eV; Collision energy potential: 22 V; Collision energy spread: 30 eV.
2. *Conjugation with Coenzyme A:* CoA-SH (2.82 mg) and TCEP (10 mM in PBS, 360 µL) were mixed and stirred at 40°C for 1 h. Solution was cooled to room temperature and aminoethyl maleimido biotin (1.32 mg) dissolved in DMF (240 µL) was added to the solution. The reaction mixture was stirred at 35°C for 4 h. When complete disappearance of the starting reagent was observed by HPLC, the crude reaction mixture was purified by RP-HPLC and the resulting product was freeze-dried. MS (ESI): predicted (m/z): [M+H]^+^: 1134.9. Found: 1134.3. MS parameters: Curtain gas 10 mL/min; Ion spray voltage: 5500 V; Temperature: not used; Declustering potential: 75 V; Entrance potential: 10 V; Collision energy: 50 eV; Collision energy potential: 43 V; Collision energy spread: 40 eV.

### Biotinylation of NGF and its receptors

15 µM purified NGF-A4 and proNGF-A4 were incubated for 40 minutes at 37°C with a reaction mix (10 mM MgCl_2_, 10 µM CoA-biotin and 1 µM Sfp Synthase (SfpS) or Acp Synthase (AcpS) (New England Biolabs), or no enzyme as control in phosphate buffer up to 30 µl final volume. Untagged proNGF and NGF were subjected to the same reaction as control.

A1/S6-tagged TrkA-EGFP and P75NTR-EGFP constructs were sequentially transfected into SH-SY5Y cells. The same constructs devoid of A1/S6-tags and the ACP-TrkA construct [4] were transfected as controls. 24 h later, cells were serum-starved for at least 2 hours and then incubated for 40 minutes at 37°C with a reaction mix prepared in serum/antibiotic-free DMEM-F12 supplemented with 0.5% BSA, 10 mM MgCl_2_, 10 µM CoA-biotin and 2 µM of SfpS or AcpS, or no enzyme as control.

### Western Blot

2 µl of all NGF/proNGF biotinylation reactions were treated under denaturing conditions (100°C, 8 minutes in 2× Laemmli Sample Buffer), run on two gels (1 µl for each gel) and electrotransferred to two PVDF membranes. These were blocked in TBST+5% w/v BSA, then one of them was blotted with anti-NGF antibody, while the other one was incubated with HRP-conjugated streptavidin (Zymed) 1∶10000 diluted in blocking solution.

Biotinylated cell monolayers were washed in ice-cold PBS and lysed in RIPA buffer supplemented with proteases and phosphatases inhibitors. 500 µg of each clarified lysate were incubated over night at 4°C with anti-TrkA or anti-P75NTR antibodies. Immunocomplexes were precipitated with Dynabeads-Protein A (Invitrogen) for at least 30 minutes at room temperature. After washing, the beads were eluted under denaturing conditions (100°C, 10 minutes in 2× Laemmli Sample Buffer), run on a gel and electrotransferred to PVDF membranes. These were washed and blocked in TBST+5% w/v BSA before incubation with HRP-conjugated streptavidin diluted (1∶5000) in blocking solution. The input lysates of the IP samples were loaded on a parallel gel and blotted against TrkA, GFP or P75NTR for normalization of the biotinylation signal. Filters were developed by electrochemiluminescence system (GH). Densitometric analysis was performed using ImageJ software (http://imagej.nih.gov/ij/). Briefly, the integrated density of the biotin signal was normalized to that of the respective GFP (for TrkA-EGFP, A1-TrkA-EGFP, S6-TrkA-EGFP lanes), TrkA (for ACP-TrkA lanes), or P75NTR (for P75NTR-EGFP, A1-P75NTR-EGFP, S6-P75NTR-EGFP lanes) band, with the higher value normalized to 1. Results reported are mean±sem from 3 (panel A) and 2 (panel B) independent blots. Obtained images were subjected to linear contrast enhancement after image analysis.

Antibodies used in the experiments were: anti-TrkA (06-574, Millipore; 1∶500); anti-P75NTR (07-476, Millipore; 1∶1000); anti-GFP (ab290, Abcam; 1∶1000); anti-NGF (sc-549, Santa Cruz Biotechnology; 1∶2000).

### TIRF imaging and data analysis

S6-TrkA and A1-P75NTR constructs cloned in TMPrtTA “all-in-one” lentiviral vector were transfected into SH-SY5Y cells. Constructs were either transfected one at a time (control experiments) or co-transfected. 5 h after transfection each transfected plate was split and cells seeded onto Willco glass-bottom dishes. Transgene expression was induced adding 0.05 µg/ml doxycycline overnight. Cells were serum starved for at least 2 hours and then incubated at 37°C with two sequential reaction mixes containing the two different PPTases. For all data shown here, the first mix contained AcpS, the second one SfpS; indeed, keeping A1 tag labeling as first minimized unspecific labeling in the control experiments. Each mix was prepared in serum/antibiotic-free DMEM-F12 supplemented with 0.5% BSA, containing 1.0 µM AcpS or SfpS, 10 mM MgCl_2_ and 10 µM biotin-CoA. The typical incubation time was 20 min for both enzymes. However we observed that reducing incubation times under 10 min (down to 5 min for AcpS and 7.5 min for SfpS) helped to minimize unspecific labeling (Fig. S3). Longer incubation times (>20 min) produced much higher non-specific signal in the control experiments. After each biotinylation step, cells were washed twice in PBS before a 6–10 min incubation at room temperature with Streptavidin-coated quantum dots (S-Qdots525 or 655, Invitrogen) in Borate buffer pH 8.3, 0.5% BSA and 215 mM sucrose as previously described [5]. 2 nM S-Qdots were used for detection of receptor single molecules, as previously reported [5]. Up to four PBS washes were necessary to remove unbound S-Qdots. Two incubations were performed in between the two PPTases labeling reactions: the former consisted in a 2 min incubation with 200 nM free streptavidin in order to saturate any biotinylated receptor eventually left uncoupled from the respective S-Qdot. Next, a 2 min incubation with 200 nM free biotin was performed, to saturate all the streptavidins introduced in the previous steps. These washes constitute the “saturate and rinse” step of the dual-color labeling protocol.

Labeled cells were immediately imaged at 37°C, 5% CO_2_ by a Leica DM6000 microscope capable of transmission DIC imaging and equipped with the Leica TIRF-AM module, an incubator chamber, an electron multiplying charge-coupled-device (EM-CCD) camera (ImagEM C9100-13, Hamamatsu), and a 100× oil immersion objective (NA 1.47). TIRF images were acquired sequentially on ROIs that included the basal membrane of cells using the 405 nm laser line and exploiting an emission fast filter wheel with Semrock FF01-525/45 and FF01-655/10 emission filters for the detection of the green and the red channels, respectively. The integration time was 20 ms for the red channel, 80 ms for the green channel. The penetration depth was set at 90 nm. Time-lapse videos were acquired for each analyzed cell, to check that observed particles in the two channels were actually moving receptors (Videos S1, S2, S3). In this case, the same experimental set-up described for the still images was used, and time lapses were acquired for ∼8.5 s with a frame time of 167 ms.

Acquired images were analyzed using ImageJ software (http://imagej.nih.gov/ij/) by counting the number of particles detected in the two channels for the single transfections and the co-transfection. The % of green and red over total particles for each cell is plotted in box chart graphs for the three experiments. We discarded from this analysis cells that displayed a density of Qdots too high for single-particle studies in one of the fields (most often in the green one, as A1P75NTR was generally more expressed than S6-TrkA at the 0.05 µg/ml doxycycline dose). Presented images were subjected to background subtraction and linear contrast enhancement after analysis.

## Results

ACP and PCP tags belong to a family of protein and peptide tags that can be covalently conjugated to virtually any small-probe-substituted PP arm of a CoA substrate by post-translational modification enzymes named PPTases [6]. Here we exploited shorter tags, derived from *in vitro* evolution studies committed to the shortening of the ACP and PCP tags [7], [8].

**Figure 1:**
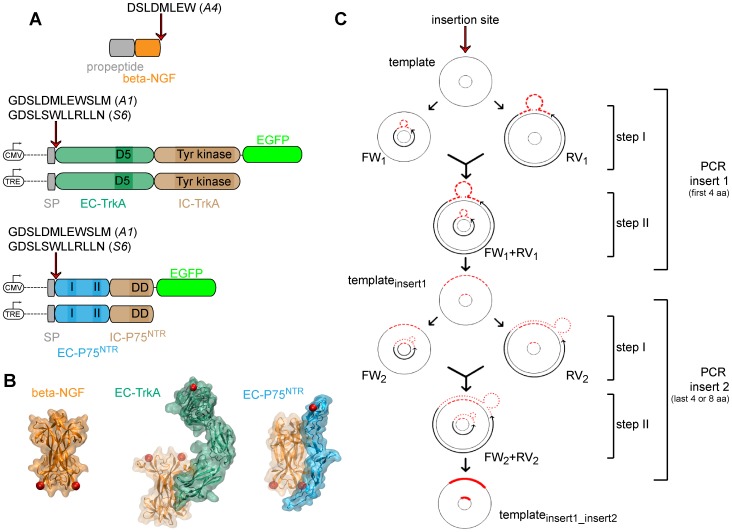
Schematic overview of the insertional mutagenesis method. **A**) Cartoon depicting NGF, TrkA, and P75NTR constructs prepared for this study. The site of tag insertion is indicated in each case by a red arrow. On top of the arrow the complete amino acidic tag sequence is reported. The promoters used for expression of the receptors are depicted upstream each receptor construct. On the extracellular domain of the receptors, sites of interaction with NGF are highlighted; on the intracellular domain of the receptors, sites of receptor activity are highlighted. Abbreviations: CMV = Cytomegalovirus promoter; TRE = Tet-Responsive-Element promoter; SP = signal peptide; EC = extracellular domain; IC = intracellular domain; D5 = proximal immunoglobulin-like domain; I = NGF-interaction site 1; II = NGF-interaction site 2; DD = death domain. **B**) Crystal structure of NGF (left; PBD. n.1BET), NGF-TrkA (EC) (middle; PBD. n.2IFG), NGF-P75NTR(EC) (right; PBD. n.1SG1). The color code is the same as in panel A. Red spots highlight the sites of tag insertion in each protein. **C**) Scheme of the insertional mutagenesis procedure used in this study. Details are provided in Text S1.

We prepared recombinant constructs encoding NGF, TrkA and P75NTR proteins (schematically depicted in Fig. 1A) by insertion of the shorter aminoacidic tags into their sequences. The A4 tag sequence [8] (Fig. 1) was inserted at the C-terminus of NGF, while A1 and S6 tags sequences [7] (Fig. 1) were inserted into the extra-cellular (EC) domains of both TrkA and P75NTR proteins, downstream of the signal peptide at the N-terminal position. As a result, the tag is exposed at the cell surface upon receptor translocation to the plasma membrane. Tag insertion sites are highlighted as red dots in the crystal structures of NGF and of the receptors EC domains in Fig. 1B. Our insertional mutagenesis procedure is schematically depicted in Fig. 1C and described in detail in Text S1. This strategy yields tag insertion in the desired sites with no need for any linker aminoacidic sequence. We thus took full advantage of the efforts that have progressively converted the ACP and PCP protein tags into the shorter A1, A4, and S6 peptide tags [7], [8]. We next examined whether these inserted peptides could be effectively functionalized by PPTase enzymes without perturbing protein functionality. We chose biotin, carried by CoA-biotin substrates, as well-known test moiety.

**Figure 2:**
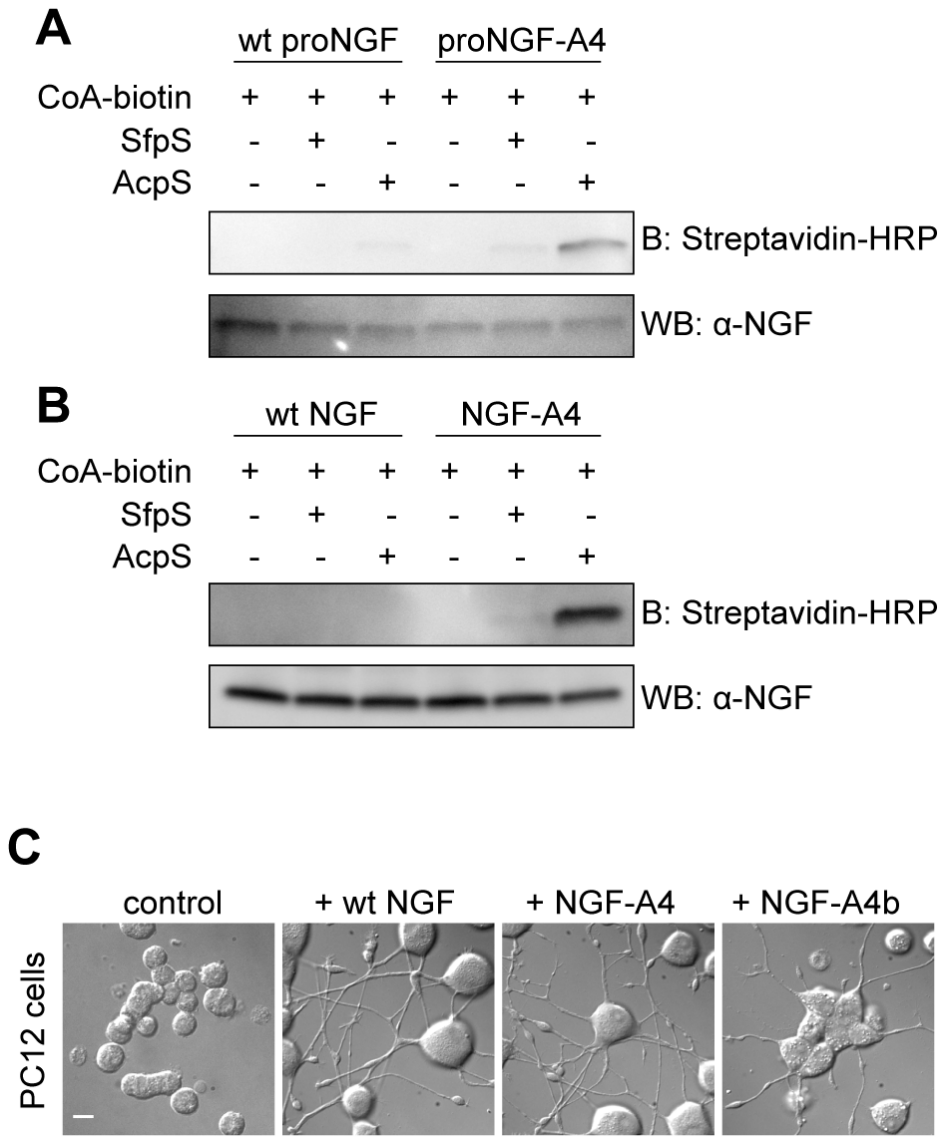
Site-specific biotinylation of proNGF and NGF. **A–B**) Western blot for the analysis of the *in vitro* biotinylation reaction of purified NGF-A4 (**A**) and proNGF-A4 (**B**) using CoA-biotin substrate and AcpS or SfpS PPTases. The same biotinylation reaction is performed in parallel using untagged wt NGF and wt proNGF as negative controls. Streptavidin-HRP is used for detection of biotin. The anti-NGF blot is the loading control. **C**) Typical DIC images obtained when performing the differentiation assay in PC12 cells using ∼50 ng/ml of wt NGF, NGF-A4 and biotinylated NGF-A4 (NGF-A4b). Untreated cells are the control. Scale bar: 20 µm.

The proNGF-A4 recombinant construct was expressed and produced in E. coli, collected from inclusion bodies and purified by FPLC ion exchange chromatography with the same procedure adopted for wt recombinant proNGF [14]. ProNGF-A4 purified protein was then digested by trypsin and further purified by FPLC ion exchange chromatography. We were thus able to obtain the purified tagged mature neurotrophin using the same protocol adopted for the wt counterpart. Purified NGF-A4 and proNGF-A4 were incubated with CoA-biotin substrate and AcpS or SfpS PPTases. The same *in vitro* biotinylation reaction was performed in parallel using untagged wt NGF and wt proNGF as controls. Western blot analyses of all biotinylation reactions are reported in Fig. 2 (panels A–B). Data show that specific biotin labeling was achieved for NGF-A4 and proNGF-A4 reacted with AcpS. In order to verify if the modified neurotrophin still maintains its biological function, we performed a differentiation assay with PC12 cells. These cells endogenously express NGF receptors TrkA and P75NTR and, when incubated with wt NGF, undergo neuronal differentiation which manifests morphologically as a neurite network. PC12 cells were incubated for 5 days with ∼50 ng/ml purified wt NGF, NGF-A4 or biotinylated NGF-A4. The last one was purified from the biotinylation reaction using desalting columns (see Methods) before addition to the cell medium. We found that both NGF-A4 and biotinylated NGF-A4 do induce PC12 differentiation to a similar extent of wt NGF, thus proving that the modified neurotrophin retains its biological activity (Fig. 2C and Fig. S2).

**Figure 3:**
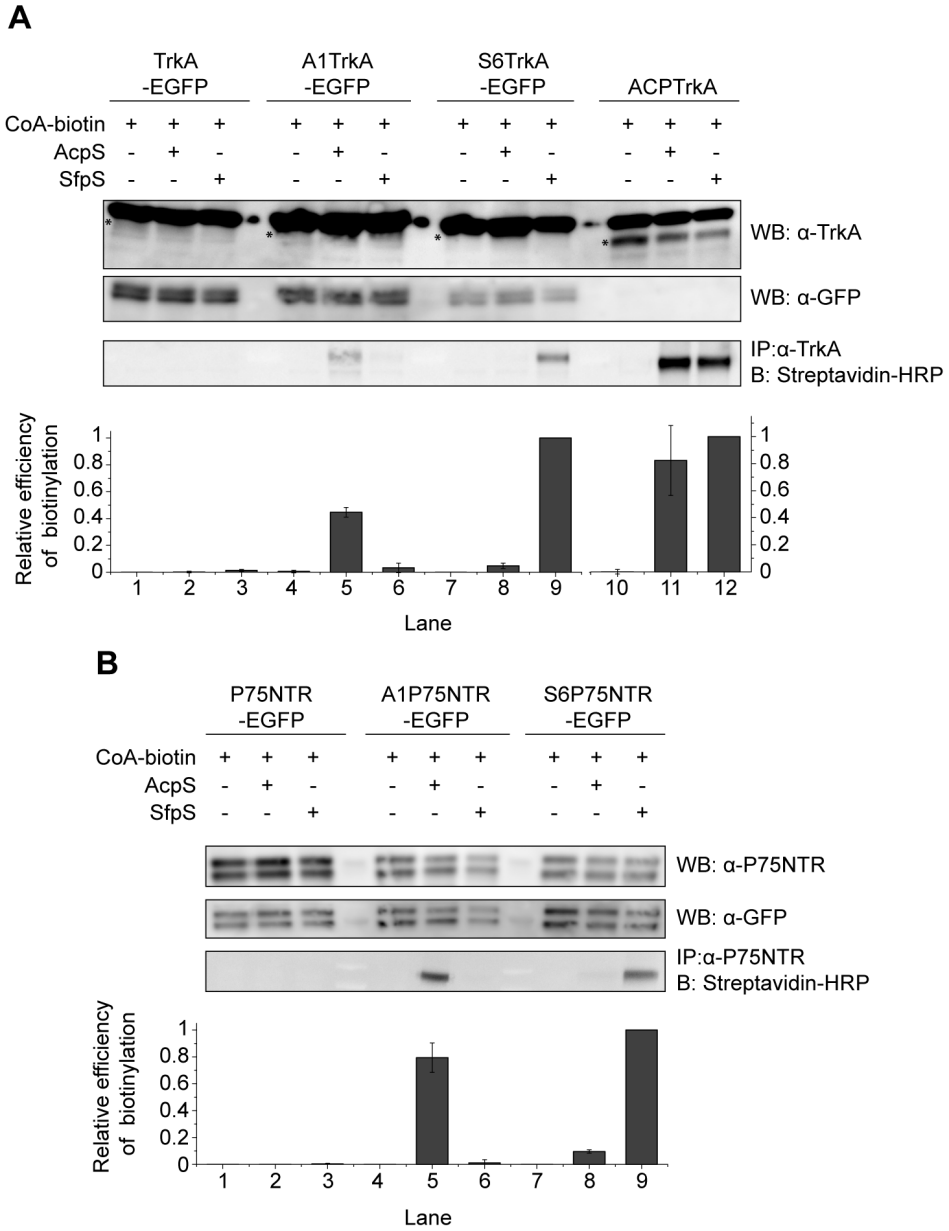
Site-specific biotinylation of TrkA and P75NTR receptors. Western blot for the analysis of the biotinylation reaction in living cells of A1/S6-TrkA-EGFP (**A**) and A1/S6-P75NTR-EGFP constructs (**B**) using CoA-biotin substrate and AcpS or SfpS PPTases. The same biotinylation reaction is performed in parallel using untagged TrkA-EGFP (**A**) and P75NTR-EGFP (**B**) as negative controls, and ACP-TrkA (**A**) as positive control. Streptavidin-HRP is used for detection of biotin. Anti-TrkA (**A**) and anti-P75NTR (**B**) blots are loading controls together with anti-GFP (both panels). The anti-TrkA blot contains an unspecific band running over TrkA, as already shown [4]; the actual TrkA band in each lane is highlighted by a star. At the bottom of each panel the densitometric analysis of the blot bands is reported. The biotin signal was normalized to the content of GFP (for TrkA-EGFP, A1-TrkA-EGFP, S6-TrkA-EGFP lanes), TrkA (for ACP-TrkA lanes), and P75NTR (for P75NTR-EGFP, A1-P75NTR-EGFP, S6-P75NTR-EGFP lanes), with the higher value normalized to 1. Results reported are mean±sem from 3 (panel A) and 2 (panel B) independent blots.

We next assessed the biotinylation performance of A1 and S6 tags inserted at the N-terminus of TrkA and P75NTR receptors. We previously demonstrated that insertion of the longer full-length ACP tag, at this position, does not hamper TrkA ability to bind NGF [4], [5]. As for P75NTR, its N-terminal region is not involved in an interaction with bound NGF [16], [17]. We used a biotinylation procedure at the surface of living cells similar to what previously reported for the ACP-TrkA construct [4], [5]. A1- and S6- TrkA-EGFP and P75NTR–EGFP constructs (as depicted in Fig. 1A) were transfected in SH-SY5Y neuroblastoma cells. 24 h post-transfection the cell monolayer was biotinylated adding CoA-biotin and either AcpS or SfpS PPTases in the cell medium. Cells were then lysed and immunoprecipitated using either anti-TrkA or anti-P75NTR antibodies. Samples were loaded on a gel and blotted using Streptavidin-HRP. Figure 3 shows that the A1 tag is specifically biotinylated by AcpS for both receptors (at least ten-fold compared to SfpS, according to the densitometric analysis); the same is true for S6 tag reacted with SfpS. Conversely, the ACP-TrkA used as a control is equally biotinylated by the two PPTases. In general, we found the A1 tag to be less efficiently labeled than the S6 tag for the same construct, especially in the case of TrkA where A1-TrkA is biotinylated about 60% less than S6-TrkA. These data prompted us to use the combination of S6-TrkA and A1-P75NTR in subsequent experiments. Taken together, these data confirm for TrkA and P75NTR in living cells, the properties of orthogonal labeling shown for A1 and S6 tags in previous *in vitro* studies [7]. Furthermore, as this procedure only allows the biotinylation of the receptor pool exposed at the cell surface, our data suggest that insertion of A1 and S6 tags downstream the signal peptide of TrkA and P75NTR receptors does not inhibit their translocation at the cell membrane.

**Figure 4:**
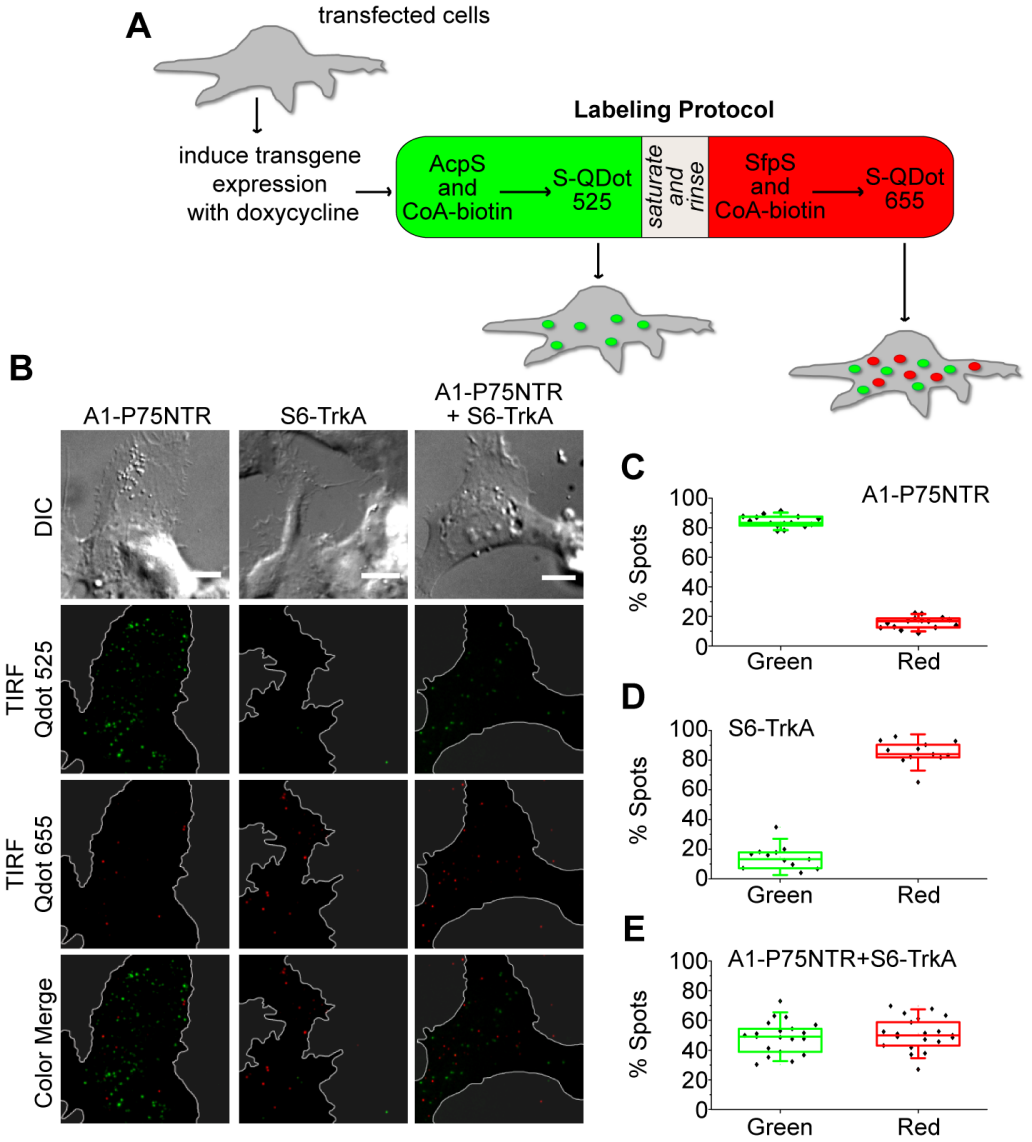
TIRFM detection of PPTase-specific A1-P75NTR and S6-TrkA fluorolabeling. **A**) Scheme of the dual-color labeling protocol used in the experiment. Details are provided in Materials and Methods. **B**) TIRF microimages analyzing PPTase-specific A1-P75NTR and S6-TrkA fluorolabeling at the SH-SY5Y plasma membrane. A1 labeling by AcpS is detected by S-Qdot525; S6 labeling by SfpS is detected by S-Qdot655. Borders of the cells basal membrane, determined through the DIC image, are highlighted in the corresponding TIRF images by a white line, while areas outside cells are grayed to simplify image interpretation. Scale bars: 10 µm. **C–E**) Quantification of the % of green and red over total particles at the basal membrane of each analyzed cell expressing A1-P75NTR (**C**), S6-TrkA (**D**) or both constructs (**E**).

We next examined whether the use of A1 and S6 tags allows the simultaneous fluorolabeling of single molecules of TrkA and P75NTR receptors when co-expressed in the same cell. SH-SY5Y cells were co-transfected with the inducible S6-TrkA and A1-P75NTR constructs (see Fig. 1A). A control transfection with either construct alone was also performed. Transgene expression was then induced using a low dose of doxycycline. This choice avoided receptor overexpression in the cells that, in turn, may have hampered single-receptor detection. Cells were subjected to a sequential dual-color staining procedure, as outlined in Fig. 4A, in order to label receptors exposed at the cell surface. In more detail, the exposed A1 tag was first biotinylated using AcpS enzyme; A1-P75NTR construct was then coupled to S-Qdot525. In the next step, exposed S6 tag was biotinylated using SfpS enzyme; S6-TrkA construct was finally coupled to S-Qdot655. Labeled cells were imaged in the two different channels by TIRF microscopy (Fig. 4B and Videos S1, S2, S3), and the number of single molecules detected in the two channels quantified for each cell (Fig. 4C–E). Cells transfected with both receptors were similarly stained with the two different S-Qdots upon labeling (Fig. 4E); single transfections performed as a control yielded labeling largely dominated by the Qdots added after the reaction with the tag-specific PPTase (Fig. 4 C and D). These data demonstrate that the use of A1 and S6 tags leads to orthogonal fluorolabeling of TrkA and P75NTR receptors co-expressed in living cells.

## Discussion

The experimental study of molecular interactions occurring between NGF (more in general, neurotrophins) and its receptors requires means to label them independently, simultaneously, and with controlled stoichiometry. In this work we developed a toolbox for this aim. We describe here a method for the introduction of three different tags into the sequence of NGF (both as immature and mature neurotrophin) and of TrkA and P75NTR receptors (Fig. 1). The chosen tags belong to the ACP and PCP families [3], [9] and bear a serine residue as the site of covalent transfer of the CoA PP arm by PPTase enzymes. In all experiments presented here, the CoA PP arm is substituted with biotin so that we achieve site-specific biotinylation of (pro)NGF and its receptors. We wish to stress, however, that virtually any small-probe carried by CoA PP arms can be coupled to the three proteins. (pro)NGF is labeled *in vitro*, after purification of the proneurotrophin expressed in E. coli (Fig. 2). On the other hand, TrkA and P75NTR are labeled in living cells that express the tagged receptors (Fig. 3). PPTases and CoA-biotin substrate are added to the cell medium and do not permeate the cell membrane, so that only the receptor pool exposed at the cell surface is actually biotinylated. Fluorolabeling of the two receptors at the cell membrane is achieved by addition of two spectrally-distinct S-Qdots to the cell medium (Fig. 4), and their subsequent visualization at the single-receptor level.

Our strategy fulfills all the recommended criteria to achieve the specific labeling of proteins of interest that are involved in molecular interactions (see Introduction). First of all these tags are small, being shortened versions of the ACP and PCP tags [7], [8]: A4 tag fused to NGF is 8 amino-acid long, while A1 and S6 tags fused to either TrkA or P75NTR are 12 amino-acid long. Our insertional mutagenesis method (Fig. 1C and Text S1, Tables S1, S2) makes it possible to insert tags with no need for any additional flanking or linker sequence in virtually any site of the protein of interest. This is particularly relevant for the case of TrkA and P75NTR, since their tag insertion site is neither the N-terminus nor the C-terminus of the receptors, but is downstream the signal of localization to the plasma membrane (Fig. 1A). Traditional tag-cloning procedures would in this case result in the insertion of additional amino acids thus compromising the effective gain resulting from tag shortening. Although the choice of not inserting any linker sequence may in principle hinder accessibility of the tags for the labeling reaction, this seems not to be the case for most of our constructs. We provided unambiguous biochemical evidence that tag biotinylation occurs both for the neurotrophin (Fig. 2) and for its receptors (Fig. 3). Nevertheless, in the latter case we experienced a reproducible lower degree of biotinylation for A1 tag than for S6 tag; as for P75NTR (Fig. 3B), such small difference can be likely ascribed to the higher enzyme efficiency reported for SfpS when compared to AcpS in the tag labeling reactions [7]. However, the unexpected ≈60% reduction of A1-TrkA compared to S6-TrkA biotinylation (Fig. 3A) hints at a possible protein environment in TrkA EC domain that hampers A1 but not S6 tag labeling by PPTases. Notably, the two tags are differently charged at physiological pH; indeed, A1 tag displays three negatively charged residues, while S6 tag bears one positively and one negatively charged residue leading to an overall neutral charge status. We are tempted to propose that possible electrostatic interactions between negatively charged A1 tag and the surrounding proteic TrkA environment affect the efficiency of tag recognition by AcpS. Further analysis are necessary to clarify this hypothesis, but the relevant conclusion, important for the methodological work presented here, is that S6-TrkA biotinylation is more efficient.

Overall, this tag-length optimization likely minimizes any interference with recombinant-protein folding and function, or with complex formation with respect to the endogenous counterparts. Importantly, the insertion sites chosen for the tags are far from residues involved in the formation of NGF-receptor complexes (Fig. 1B) and were already reported to lead to functional proteins, at least for the case of NGF [18] and TrkA [4].

Generally, non-specific biotinylation of proteins is achieved by chemical conjugation of reactive biotin derivatives to amine, thiol or carboxyl groups of proteins [19]. The main disadvantage of this approach is the lack of control in number and type of biotinylated sites of the target proteins, so that mixed populations of labeled proteins are obtained; this can potentially lead to impairment of biological activity and lack of experimental reproducibility. Our choice of inserting A1, A4 and S6 tags ensures 1∶1 stoichiometry between the labeled neurotrophin/receptor and biotin. This is an important aspect from the point of view of microscopy and in view of tracking individual membrane proteins (like TrkA and P75NTR) (or their complexes) in living cells [20]. It may even allow the determination of complex stoichiometry. Also, we wish to underline the relevance of the present approach for its application to NGF and in general neurotrophins. In most of the papers reported to date, neurotrophins were chemically coupled to biotin [21], [22], [23], [24] and organic fluorophores [25], [26], [27], [28], leading to mixed populations containing 3–9 small probes per neurotrophin depending on the experimental procedure used. The possibility presented here of labeling NGF with 1∶1 stoichiometry will yield more reproducible results and is optimal for single-molecule imaging. In this context, the performance of our mono-functionalized NGF will be similar to what recently reported for NGF-AVI tag construct [18]. We should like to point at one significant advantage of the present approach over the AVI (AP) tag/biotin ligase system: any substituted PP arm of CoA substrates can in principle be fused to the protein of interest, besides (and in addition to) the biotinylated one [6]. We therefore envisage the possibility of broadening the spectrum of applications for this recombinant neurotrophin, from standard biochemistry to single-molecule imaging and counting, from electron microscopy to NMR studies depending on the probe used for NGF labeling.

Finally, in this work we report the possibility of simultaneous individual imaging of the two neurotrophin receptors (Fig. 4). This is achieved by introduction of A1 and S6 tags in the sequences of P75NTR and TrkA, respectively. Such tags were already reported to display properties of orthogonal labeling *in vitro* and in living cells by using two different PPTases [7]. Here we demonstrate the possibility of detecting by TIRF microscopy single molecules of the two tagged receptors labeled by two different S-Qdots in living cells. This is made possible by controlling (and restraining) the level of expression for the tagged receptors in the cells using Tet-ON inducible expression systems and by adopting an optimized dual-color staining method (Fig. 4A and Materials and Methods section) to minimize the cross-reaction due to the use of biotin-streptavidin interaction for both A1 and S6 tags. Under these conditions, we demonstrated at least ∼85% PPTase-specific fluorolabeling for both receptors, as quantified by counting the number of single particles detected in the green and red channels in cells transfected with the two receptors separately (Fig. 4 C and D). We believe that the residual unspecific labeling does not represent a real concern when performing dual-color experiments (see also Fig. S3). First of all one must note that this percentage is unavoidably an overestimate owing to Qdots aspecifically adsorbed at the basal membrane. These are present in both channels but obviously have a more sizable impact on the estimated percentage in the less populated channel. More importantly we stress that the aspecificity can be further reduced by shortening the PPTase incubation times during the dual-color labeling procedure. In conclusion we believe that these results represent a viable method to simultaneously image and track TrkA and P75NTR receptors in the same cell by using two different PPTases and two spectrally-distinct fluorophores.

## Supporting Information

Figure S1 **Yields of production of proNGF-A4 and NGF-A4** ***versus*** **wt proNGF and NGF.** The production yield is expressed as quantity of obtained purified protein (in mg) per liter of bacterial culture volume. Mean values obtained are represented by histogram bars. Error bars represent standard deviations of four and three independent productions of proNGF-A4 and NGF-A4, respectively.
(TIF)

Figure S2 **Quantitative morphometric analysis of differentiated PC12 cells.** The distributions of *Diff* (% of differentiated cells in a field, panel **A**) and av.neurites/cell (average number of neurites per differentiated cell, panel **B**) for the 4 samples analyzed (untreated control: n = 11 fields comprising 147 cells, NGF-A4: n = 18 fields comprising 138 cells, NGF-A4b: n = 9 fields comprising 52 cells, wt NGF: n = 15 fields comprising 139 cells) are reported as mean±sem. Statistical analysis was performed using the one-way ANOVA test, to compare the distributions of NGF-A4 and biotinylated NGF-A4 (NGF-A4b) to the same obtained for the control (ctrl) and wt NGF (wt). Obtained P values below 0.05 were considered significant (*), vice versa they were considered not significant (#).
(TIF)

Figure S3 **Dependence of the specificity of A1P75NTR labeling on the presence of immobile, adsorbed Qdot under the plasmamembrane and on the duration of PPTase labeling reactions.** Quantification of the % of green and red over total particles at the basal membrane of each analyzed cell expressing A1-P75NTR, upon (left to right): 1) 20 min PPTase incubation in the labeling reaction (same data of Fig. 4C); 2) same data as 1, corrected for the number of aspecific (immobile, probably blocked on the glass) Qdots under the plasma membrane in the two channels, as estimated considering the density of immobile Qdots at the basal membrane of non-transfected cells; 3) data obtained from cells labelled using PPTase incubation times shorter than 10 min (∼5 min for AcpS and ∼7.5 min SfpS).
(TIFF)

Table S1 **List of insertional primers used for constructs preparation.**
(DOCX)

Table S2 **Scheme of the PCR program used for the insertional mutagenesis.**
(DOCX)

Text S1 **Supporting information for the insertional mutagenesis protocol.**
(DOCX)

Video S1 **Time-lapse TIRF imaging of A1-P75NTR at the basal membrane of a cell subjected to the dual-color labeling protocol.**
(AVI)

Video S2 **Time-lapse TIRF imaging of S6-TrkA at the basal membrane of a cell subjected to the dual-color labeling protocol.**
(AVI)

Video S3 **Time-lapse TIRF imaging of A1-P75NTR and S6-TrkA at the basal membrane of a cell subjected to the dual-color labeling protocol.**
(AVI)
