# Supplementary figures and images for: Site-Specific Labeling of Neurotrophins and Their Receptors via Short and Versatile Peptide Tags
Source: PLoS One. 2014 Nov 26;9(11):e113708. doi: 10.1371/journal.pone.0113708 (PMC4245215; doi:10.1371/journal.pone.0113708)

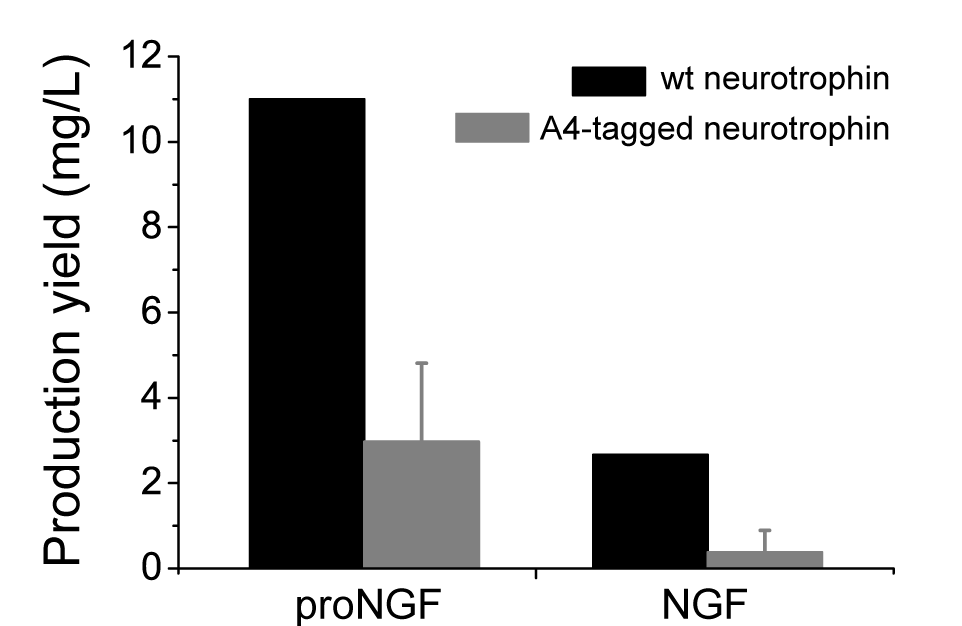

Supplement: Figure S1 — Yields of production of proNGF-A4 and NGF-A4 versus wt proNGF and NGF. The production yield is expressed as quantity of obtained purified protein (in mg) per liter of bacterial culture volume. Mean values obtained are represented by histogram bars. Error bars represent standard deviations of four and three independent productions of proNGF-A4 and NGF-A4, respectively. (TIF) [file pone.0113708.s001.tif]

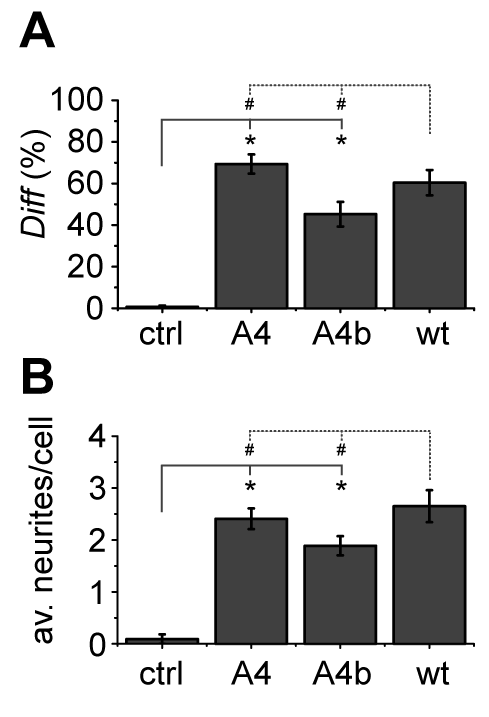

Supplement: Figure S2 — Quantitative morphometric analysis of differentiated PC12 cells. The distributions of Diff (% of differentiated cells in a field, panel A) and av.neurites/cell (average number of neurites per differentiated cell, panel B) for the 4 samples analyzed (untreated control: n = 11 fields comprising 147 cells, NGF-A4: n = 18 fields comprising 138 cells, NGF-A4b: n = 9 fields comprising 52 cells, wt NGF: n = 15 fields comprising 139 cells) are reported as mean±sem. Statistical analysis was performed using the one-way ANOVA test, to compare the distributions of NGF-A4 and biotinylated NGF-A4 (NGF-A4b) to the same obtained for the control (ctrl) and wt NGF (wt). Obtained P values below 0.05 were considered significant (*), vice versa they were considered not significant (#). (TIF) [file pone.0113708.s002.tif]

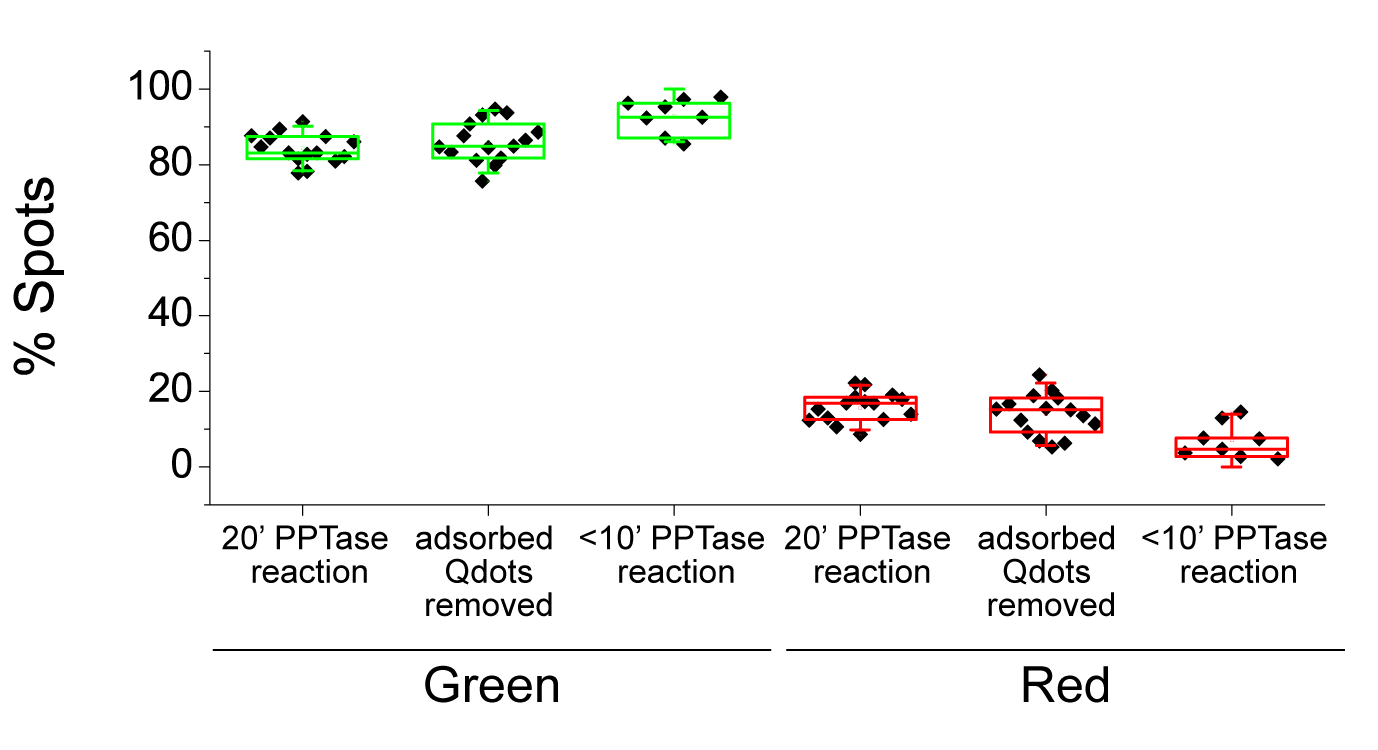

Supplement: Figure S3 — Dependence of the specificity of A1P75NTR labeling on the presence of immobile, adsorbed Qdot under the plasmamembrane and on the duration of PPTase labeling reactions. Quantification of the % of green and red over total particles at the basal membrane of each analyzed cell expressing A1-P75NTR, upon (left to right): 1) 20 min PPTase incubation in the labeling reaction (same data of Fig. 4C); 2) same data as 1, corrected for the number of aspecific (immobile, probably blocked on the glass) Qdots under the plasma membrane in the two channels, as estimated considering the density of immobile Qdots at the basal membrane of non-transfected cells; 3) data obtained from cells labelled using PPTase incubation times shorter than 10 min (∼5 min for AcpS and ∼7.5 min SfpS). (TIFF) [file pone.0113708.s003.tiff]
