# Supplementary material for: Site-Specific Labeling of Neurotrophins and Their Receptors via Short and Versatile Peptide Tags
Source: PLoS One. 2014 Nov 26;9(11):e113708. doi: 10.1371/journal.pone.0113708 (PMC4245215; doi:10.1371/journal.pone.0113708)
Supplement: Table S1 — List of insertional primers used for constructs preparation. (DOCX) [file pone.0113708.s004.docx]

*Table S1. List of insertional primers used for constructs preparation.* The inserted sequence coding for A4, A1 or S6 tags is reported in capital letters in each primer sequence. Each FW and RV pair is formed by complementary primers.

| **Primer name and length** | **Primer sequences (5’→3’)** |
| --- | --- |
| proNGF-A4*insert*-FW (72mer) | ctctctagaaaggctgtgagagattctcttgattctcttgatatgttggagtggtgataaggatccggctgc |
| proNGF-A4*insert*-RV (72mer) | gcagccggatccttatcaCCACTCCAACATATCAAGAGAATCaagagaatctctcacagcctttctagagag |
| proNGF-A4*insert1*-FW (56mer) | gctctctcagaaaggctgtgagaGATTCTCTTGATtgataaggatccggtgctaac |
| proNGF-A4*insert1*-RV (56mer) | gttagcagccggatccttatcaATCAAGAGAATCtctcacagcctttctagagagc |
| proNGF-A4*insert2*-FW (56mer) | ggctgtgagagattctcttgatATGTTGGAGTGGtgataaggatccggctgataac |
| proNGF-A4*insert2*-RV (56mer) | gttagcagccggatccttatcaCCACTCCAACATatcaagagaatctctcacagcc |
| TrkA-*insert1*-FW (56mer) | ggctgatactggcatctgcgggcGGAGATTCTCTTgccgcaccctgccccgatgcc |
| TrkA-*insert1*-RV (56mer) | ggcatcggggcaggggtcggcAAGAGAATCTCCgcccgcagatgccagtatcagcc |
| TrkA-A1*insert2*-FW (66mer) | tctgcgggcggagattctcttGATATGTTGGAGTGGTCTTTGATGgccgcaccctgccccgatgcc |
| TrkA-A1*insert2*-RV (66mer) | ggcatcggggcagggtgcggcCATCAAAGACCACTCCAACATATCaagagaatctccgcccgcaa |
| TrkA-S6*insert2*-FW (66mer) | tctgcgggcggagattctcttTCGTGGCTGCTTAGGCTTTTGAATgccgcaccctgccccgatgcc |
| TrkA-S6*insert2*-RV (66mer) | ggcatcggggcagggtgcggcATTCAAAAGCCTAAGCAGCCACGAaagagaatctccgcccgcaga |
| P75NTR-*insert1*-FW (54mer) | ggggtgtcccttggaggtgccGGAGATTCTCTTaaggaggcatgccccacaggc |
| P75NTR-*insert1*-RV (54mer) | gcctgtggggcatgcctccttaagagaatctccggcacctccaagggacacccc |
| P75NTR-A1*insert2*-FW (66mer) | ggaggtgccggagattctcttGATATGTTGGAGTGGTCTTTGATGaaggaggcatgccccacaggc |
| P75NTR-A1*insert2*-RV (66mer) | gcctgtggggcatgcctccttcatcaaagaccactccaacatatcaagagaatctccggcacctcc |
| P75NTR-S6*insert2*-FW (66mer) | ggaggtgccggagattctcttTCGTGGCTGCTTAGGCTTTTGAATaaggaggcatgccccacaggc |
| P75NTR-S6*insert2*-RV (66mer) | gcctgtggggcatgcctccttattcaaaagcctaagcagccacgaaagagaatctccggcacctcc |
