# Supplementary material for: Site-Specific Labeling of Neurotrophins and Their Receptors via Short and Versatile Peptide Tags
Source: PLoS One. 2014 Nov 26;9(11):e113708. doi: 10.1371/journal.pone.0113708 (PMC4245215; doi:10.1371/journal.pone.0113708)
Supplement: Table S2 — Scheme of the PCR program used for the insertional mutagenesis. (DOCX) [file pone.0113708.s005.docx]

*Table S2. Scheme of the PCR program used for the insertional mutagenesis*

| **PCR program** | | **Temperature** | **Time** |
| --- | --- | --- | --- |
| **Initial Denaturation** | | 95°C | 1 min |
| **Step 1**  **(10 cycles)** | **Denaturation** | 95° | 1min |
|  | **Primer annealing** | 55° | 1min 30sec |
|  | **Extension** | 68°C | 1min per Kb DNA template |
| **Step 2**  **(18 cycles)** | **Denaturation** | 95° | 1min |
|  | **Primer annealing** | 55° | 1min30sec |
|  | **Extension** | 68°C | 1min per Kb DNA template |
| **Hold** | | 4°C | ∞ |
