# Supplementary material for: Site-Specific Labeling of Neurotrophins and Their Receptors via Short and Versatile Peptide Tags
Source: PLoS One. 2014 Nov 26;9(11):e113708. doi: 10.1371/journal.pone.0113708 (PMC4245215; doi:10.1371/journal.pone.0113708)
Supplement: Text S1 — Supporting information for the insertional mutagenesis protocol. (DOCX) [file pone.0113708.s006.docx]

**Supporting Information**

*Insertional mutagenesis protocol*

We used mostly reagents and reactions of the QuickChange® XL Site-Directed Mutagenesis kit (Agilent Technologies) but adopted a modified protocol. All primer pairs used were purchased from Sigma-Aldrich purified by PAGE, and are listed in Table S1.

Each insertion was obtained by performing the following PCR reaction in 50 µl reaction: 1.5X Turbo DNA Polymerase Buffer, 250 ng insertional Primer, 1mM dNTPs, 3 µl Quick Solution, 1 µl Turbo DNA Polymerase and 15 ng DNA template. To avoid primer self-annealing artifacts, each PCR run consisted in two steps (reported in Table S2 and depicted in Fig. 1B of the main text): in the first step (10 cycles), two independent 50 µl PCR reactions were run for the same template, one containing only the FW insertional primer, the other one containing only the respective RV insertional primer. In the second step (18 cycles), the two separate reactions were mixed and two tubes containing each 25 µl of FW and 25 µl of RV reaction were supplemented with 0.75 µl Turbo DNA Polymerase. Afterwards, each 50 µl PCR reaction was subjected to digestion with 1 µl DpnI (New England Biolabs), at 37°C for 2-3h in the presence of the specific DpnI buffer to eliminate parental DNA template. The final 100 µl reaction was recovered and purified using the QIAquick PCR Purification Kit (Qiagen), eluted in 25 µl EB buffer and ~1/5 of recovered DNA was transformed into chemically competent XL10 Gold bacteria.

To obtain A1 and S6 tags inserted into the sequences of TrkA and P75NTR, the insertion of the complete tag did not lead to positive clones. Therefore, we performed two sequential PCR reactions with two partially overlapping primer pairs to introduce the first 4 (*insert1*, see Table S1) and the other 8 amino acids (*insert2*, see Table S1). Each PCR run was followed by DpnI digestion, PCR cleanup and transformation in XL10 Gold bacteria. Bacteria transformed with *insert1* reaction were grown in 5ml LB medium supplemented with antibiotic overnight at 37°C; next morning DNA was mini-prep extracted, quantified and used as template of the *insert2* PCR run. Bacteria were plated for screening only after transformation of *insert2* reaction. Colonies were picked and positive clones screened by DNA sequencing. For the insertion of A4 tag downstream the sequence of proNGF, we tried both the insertion of the complete tag (8 amino acids) with a unique primers pair and the sequential insertion of 4+4 amino acids. In the two strategies, we found the insertion yields to be 33% and 17%, respectively. Both primers pairs are reported in Table S1.

We found the efficiency of tag insertion to be strictly dependent on the total length of DNA template, with 10% efficiency displayed by TrkA-EGFP template (8719 bp), 12.5% efficiency for P75NTR-EGFP template (8078 bp), and 33% efficiency for proNGF template (6346bp).
